# A comparative analysis of the prevalence and intensity of schistosomiasis and soil-transmitted helminth infections between preschool-aged children and school-going children in KwaZulu-Natal Province

**DOI:** 10.1007/s00436-025-08504-1

**Published:** 2025-06-02

**Authors:** Tafadzwa Mindu, Nathan Chanhanga, John Mogaka, Moses Chimbari

**Affiliations:** 1 School of Nursing and Public Health, College of Health Sciences, University of KwaZulu Natal, Durban, 4001 South Africa; 2 Department of Psychiatry, Social & Behavioral Sciences, Faculty of Medicine, National University of Science and Technology, Bulawayo, 40000 Zimbabwe

**Keywords:** Schistosomiasis, Soil-transmitted helminths, Preschool-aged children, School-going children, Public health policy

## Abstract

The World Health Organization (WHO) recommends conducting a baseline survey to quantify the infection burden of schistosomiasis and soil transmitted helminths (STH) in pre-school aged children (PSAC) and school-aged children (SAC) before implementing a schistosomiasis mass drug administration intervention. The objective of the study was to compare the prevalence and intensity of schistosomiasis and soil-transmitted helminth (STH) infections between preschool-aged children (PSAC) and school-age children (SAC) in the province of KwaZulu-Natal, South Africa. The study was conducted in the province of KZN. The target population was PSAC and SAC, with a sample size of 2000 children (1176 primary school-aged children and 824 pre-school-aged children). Ethical clearance was obtained from the Biomedical Research Ethics Committee of the University of KwaZulu-Natal; informed consent from parents/legal guardians and verbal assent from SAC were solicited. Data collection involved collecting stool and urine samples from children. The prevalence and intensity of infections were compared between PSAC and SAC, using statistical methods to assess differences. The results showed that 49 (4.2%) SAC were positive for *Schistosoma haematobium*, while only 3 (0.41%) PSAC were infected. The total number of STH infections among participants was 281 (22%), with 91 in PSAC and 190 in SAC. The chi-square test showed that SAC were infected with schistosomiasis more than PSAC counterparts in the same location. However, the difference in STH prevalence between PSAC and SAC was not statistically significant, suggesting that there was not much of a difference in the prevalence of STH among SAC and PSAC. Among the STH infections, Taenia was the most prevalent, affecting approximately 20.5% of SAC and 23.4% of PSAC. This species accounted for a substantial proportion of the overall STH burden in both age groups. The study concluded that while SAC has a higher overall prevalence, a real burden exists among PSAC indicating the need to include them in MDA programs targeting *S. haematobium* in the province.

## Introduction

Globally, an estimated 230 million individuals are afflicted by schistosomiasis, while 1 billion people suffer from STH infections, with more than 90% of these cases occurring in sub-Saharan Africa (Midzi et al. 2014). In South Africa, 25 million people are at risk of contracting schistosomiasis, and each year approximately 5.5 million new cases are recorded (Lai et al. 2015). Schistosomiasis affects six of the nine provinces in South Africa, namely KwaZulu-Natal, Limpopo, Mpumalanga, Gauteng, North West and the Eastern Cape (Kabuyaya et al. 2017). A similar pattern is observed for STH. The high prevalence of schistosomiasis and STH is closely associated with poverty, poor environmental hygiene and impoverished health facilities (Hotez, Bundy et al. 2006, Senghor et al. 2014; Opara et al. 2021).

Schistosomiasis and soil-transmitted helminths (STH) are among the most prevalent neglected tropical and subtropical diseases (Bowie et al. 2004; Mutapi et al. 2011). Schistosomiasis is caused by parasitic trematodes of the genus *Schistosoma*, with three primary species affecting humans: *S. haematobium*, *S. mansoni* and *S. japonicum* (Knopp et al. 2013). The most prevalent soil transmitted helminth (STH) are *Ascaris lumbricoides, Trichuris trichiura* and the hook worms (*Ancylostoma and Necator americanus*) (Kirwan et al. 2009). Schistosomiasis and STH inflict a significant burden on the world’s poorest populations living in rural or deprived urban settings (Hotez and Kamath 2009).

The most vulnerable groups affected by schistosomiasis and STH are pre-school age children (PSAC), school-age children (SAC), women of childbearing age and individuals engaged in activities involving contact with contaminated water, such as fishermen, irrigation farmers and domestic chores (Mutsaka-Makuvaza et al. 2019; Jeza et al. 2022; Phillips et al. 2022). These infections adversely affect adults’ work capacity and fitness while stunting the growth and reducing the learning ability of children (De Sanctis et al. 2021).

Despite WHO recommendations to include PSAC in MDA programs (Organization 2020), data on infection burden in this age group remain scarce, particularly in South Africa, where MDA programs are still in the planning stages. There are still issues that need to be dealt with including accessing the children especially those not in Early Childhood Development Centres (ECDs) and limitations associated with intake of praziquantel in its solid form. The introduction of paediatric praziquantel makes it imperative to establish the distribution and intensity of schistosomiasis infections among the PSAC to inform plans for delivery of the drugs. However, this drug is not yet up for donation making it inaccessible to the most needy children in Africa (European Medicines Agency (EMA), n.d.). South Africa has yet to roll out its MDA programme for schistosomiasis.

Although WHO’s roadmap for neglected tropical diseases (2021–2030) emphasizes the importance of integrating MDA into broader public health efforts; South Africa is still in the planning and piloting phases. Taking advantage of the planning stage, the data on the burden of disease both for PSAC and SAC could help in making sure that objective decisions are made.

The hypothesis for this study is that schistosomiasis and STH infection intensity and prevalence are similar among SAC and PSAC, since they are both exposed to the same risky water contact activities. This hypothesis was based on the premise that PSAC accompany their mothers to the water bodies and are often exposed to the same infected water that the SAC are exposed to when they play in the water bodies. Our study therefore determined and compared the prevalence of schistosomiasis and STH between SAC and PSAC in KwaZulu-Natal. We also compared the differences in egg intensity for infections in both groups. The overarching research question guiding the study was: is there a difference in the burden of schistosomiasis and STH infection between preschool-aged children (PSAC) and school-age children (SAC) in the province of KwaZulu-Natal? This central research question was further broken down into specific inquiries: What is the difference in the prevalence of schistosomiasis and STH between PSAC and SAC in the province of KwaZulu-Natal, South Africa?; What is the difference in the intensity of schistosomiasis and STH between PSAC and SAC in the province of KwaZulu-Natal, South Africa? By addressing these research questions, the study sought to shed light on the comparative burden of schistosomiasis and STH infections between PSAC and SAC in KwaZulu-Natal.

## Materials and methods

### Study area

The study was conducted in the KwaZulu-Natal (KZN) province of South Africa. KZN has 11 districts, covering a total area of 94,362 km^2^ with a population of 10.7 million. The province experiences a tropical to subtropical climate characterized by a hot and wet summer (Nov. to Feb.) and a cool and dry winter (June to August). Most of the districts in the province have very poor communities with limited access to water sanitation hygiene (WASH) facilities; these conditions contribute to the spread of infectious diseases like schistosomiasis and soil-transmitted helminths (Mulopo and Chimbari 2021).

### Ethics and gate keepers’ permission

The study protocol was approved by the Biomedical Research Ethics Committee of the University of KwaZulu-Natal (BE429/19). Parents or legal guardians, village leaders, education and district authorities and participating children were informed about the purpose and procedures of the study. Written informed consent was obtained from parents or legal guardians for their children’s participation, and in addition to this, all SAC provided verbal assent. To ensure confidentiality, all parasitological findings were anonymized through coding. Children infected with schistosomiasis or/and STH were treated with praziquantel (for schistosomiasis) using the dose pole to determine dose and with albendazole (for STH).

### Study design and sampling

This study was a cross-sectional study targeting PSAC and SAC in the whole province of KZN. Data was collected between 2020 and 2021. The sample size was calculated using the Krejcie and Morgan formula for calculating a sample size to represent a population with unknown disease prevalence. To estimate the burden of schistosomiasis and soil transmitted helminth infection among pre-school age in KwaZulu-Natal province of South Africa, assuming 95% confidence and an acceptable margin of error of 5% and maximum variability, i.e., 50% (given unknown previous prevalence), a sample size of 120 subjects per district was required. The sample was further increased by a margin of 15% to account for potential non-response and multiplied by a design effect (D) of 1.5. Hence, the final sample size of the study was 150 PSAC and 150 SAC per district. Increasing the sample size reduced the type I and type II errors as well as known and unknown confounder effects. Hence, power of the sample (1-$\beta$) and (the % chance of detecting difference) of the study was set at 80%.

For each district we aimed to sample at least of 150 PSAC and 150 SAC. To achieve this, we selected 8 schools (taking 8 primary schools and 8 preschools) per district. We systematically sampled using a sampling tool accessed online (RANDOM.ORG — List Randomizer, n.d.). We selected 8 schools per district using the KwaZulu-Natal Department of Basic Education list of primary schools. We then identified an ECD nearer to the school to recruit the PSAC age group. We did not include children who were at home (non-school going); we only recruited children that where either in a pre-school, ECD centre, or primary school. In some instances there was no pre-school nearby and in such cases we would recruit learners in the ECD class within the schools identified. eThekwini district was divided into two districts by the KwaZulu-Natal Department of Basic Education, hence in our study, we considered Umlazi and Pinetown as sub-districts and sampled 8 schools in each of them.

### Specimen collection and processing

All children were provided with two containers for stool and urine collection, and parents or caregivers of PSAC were invited to assist in collecting samples. Only children with signed consent forms participated in the study. The team labelled specimen containers with unique codes and filled information on an Excel form for each child.

For *S. haematobium*, we collected urine from participants using a 100-ml plastic bottle container. Using a syringe, we extracted and processed 10 ml of the urine sample by syphoning it through a 25-mm diameter polycarbonate filter membrane (NucleoporeTM filter). Eggs were stained with 50% Lugol’s iodine saline solution and counted by two microscopists. The infection intensity was quantified as eggs per 10 ml of urine under a light microscope (Mott et al. 1982).

*S. mansoni* and STH were examined using the Kato-Katz thick smear technique (Katz et al. 1972). To view the stool sample under a microscope, the procedure involved placing a labelled glass slide and a 41.7-mg plastic template on top. To ensure even distribution of eggs and clear view of eggs, the faecal sample was placed on a nylon screen and pressed on top, to remove solid particles that would block the view of eggs. The sieved faecal material was scraped through the screen and then placed inside the hole of the plastic template. The template was then removed, leaving the faecal sample caked on the slide. One piece of the cellophane, which has been soaked overnight in methylene blue glycerol solution was placed over the faecal sample (Hong et al. 2003). Then, a clean slide was placed over the top and pressed evenly downwards to spread the faeces in a circle. The slide was carefully removed by gently sliding it sideways to avoid separating the cellophane strip. The slide with the cellophane was placed upwards under a microscope and examined in a systematic zig zag pattern. The number and type of egg of each species was recorded on the recording form alongside the same number. Finally, the number of eggs was multiplied by 24 to give the number of eggs per gram (Hong et al. 2003).

The template was placed in a bucket of water mixed with detergent, and a piece of cellophane is placed over the faecal sample. The number and type of egg of each species were recorded on the recording form. Thick faecal smears were then observed within 1 h using a light microscope. The slides will be left to clear for 24 h.

### Data analysis

During the sample collection stage, participant data was collected, including age, sex, grade, school and district. Each child was assigned a code, and this code was labelled on specimen bottles. Data was recorded in an Excel form, and microscopy results were recorded against the code for each sample. These codes were matched with the children’s details, and the reports including egg counts for schistosomiasis and STH.

The quantitative data was cleaned and imported to SPSS version 20 as well as DataTab for statistical analysis. Data cleaning involved coding responses, names, gender and infection statuses of participants, identifying missing data and removing incomplete entries and outliers. Data outliers were resolved using capping, transformation and removal. Consistency checks included data type validation, range checks, duplicate removal and cross-validation to ensure each column contained the correct data type; values fell within expected range, and unique entries. Cross-validation checks were conducted for consistency across related fields. Descriptive analysis including frequency, mean and percentage was used to summarize the demographic characteristics of the study participants.

### Variables

Prevalence was defined as the percentage number of individuals positive for any of the infections over the number that submitted samples for examination. Infection intensity was defined as the average number of egg counts expressed as arithmetic mean eggs per gram of faeces (epg). Schistosomiasis infections (prevalence) as well as STH infection and intensity were the dependent variables, while the pupil grade (categorised as PSAC and SAC), district and other demographic factors such as age and sex were the independent study variables.

### Statistical tests

Prevalence and average intensity of infections were calculated for STH and *S. haematobium (no S. mansoni was recorded)*. Confidence intervals (CIs) of 95% were obtained using binomial and negative binomial regression models, respectively, considering clustering at school or village levels. We used the negative binomial model since the distribution of egg counts was over dispersed. Further, we compared the prevalence between PSAC and SAC using the chi-square test for difference in prevalence. We also used the *z*-test statistic for two-sample test of proportions reporting the *z*-test statistic and the associated *p*-values.

Screening of STH eggs was based on a 41.7 mg Kato Katz template to determine the parasite’s egg per gram in the stool (EPG) by calculating the number of eggs counted multiplied by 24. Table 1 shows how STH intensities were categorised in accordance with WHO standards.

**Table 1:** STH intensities, categorised by on WHO standards

	Light	Moderate	Heavy
*Ascaris*	1–4999	5000–49,999	≥ 50,000
*Trichuris*	1–999	1000–9999	≥ 10,000
Hookworm	1–1999	2000–3999	≥ 4,000

The study used chi-square test to analyse variables, calculated the geometric mean intensity of parasite EPG in stool for infected and non-infected individuals and used independent samples *t*-test to compare STH infection intensity between PSAC and SAC. Bivariable analysis was performed to examine the association between independent and dependent variables, with a *p*-value of < 0.05 considered statistically significant.

The study categorized common STH infections into light, moderate and heavy infection intensity using WHO standard procedures. Experienced medical practitioners and laboratory technologists performed the procedures, and some stool samples were randomly selected for quality control.

## Results

### Socio-demographic characteristics of study participants

A total of 2000 children participated in the study; 1176 school-aged children (SAC) and 824 pre-school aged children (PSAC). The age range was 1–15 years. PSAC average age was 5 (range 1–6), while SAC average age was 9 (range 8–11). Some learners failed to produce stool samples; hence, the total for STH samples was 1275 (459 PSAC = and 816 SAC). Details on demographic characteristics are in Table 2. About half (*n* = 1012; 50.6%) of the study participants were females and (*n* = 988 − 49.4%) males. The number of school-age children (SACs) was (*n* = 1176 − 58.1%), while (*n* = 838 − 41.9%) were preschool-aged children (PSAC). There were no significant differences between PSAC and SAC participants in terms of gender (X^2^ = 2.371, *p* = 0.124).

**Table 2:** Demographic characteristics of the study participants

		S. haem (n = 2000)			STH (n = 1275)		
		Samples (n)	Positive (n)	Prevalence (%)	Samples (n)	Positive (n)	Prevalence (%)
Gender	Females	1012	26	2.6%	638	145	22.7%
	Males	988	26	2.6%	637	136	21.4%
Grade	PSAC	824	3	0.4%	459	91	19.8%
	SAC	1176	49	4.2%	816	190	23.3%
	1	2	0	0.0%	1	0	0.0%
	2	8	0	0.0%	2	0	0.0%
	3	25	0	0.0%	10	2	20.0%
	4	86	2	2.3%	42	8	19.0%
	5	528	1	0.2%	305	60	19.7%
	6	190	1	0.5%	110	21	19.1%
	7	60	1	1.7%	47	10	21.3%
	8	433	9	2.1%	309	68	22.0%
	9	516	28	5.4%	343	9	26.8%
	10	123	5	4.1%	83	15	18.1%
	11	22	4	18.2%	19	3	15.8%
	12	4	0	0.0%	3	1	33.3%
	13	2	1	50.0%	1	1	100.0%
	15	1	0	0.0%			
Age							
District	*Amajuba*	261	0	0.0%	100	14	14%
	*iLembe*	124	4	3.2%	91	7	8%
	*King Cetshwayo*	170	5	2.9%	130	18	14%
	*Ethekwini (Pinetown)*	200	3	1.5%	122	64	52%
	*Sisonke*	151	0	0.0%	109	10	9%
	*Ugu*	160	15	9.4%	124	12	10%
	*Umgungundlovu*	152	5	3.3%	68	2	3%
	*Ethekwini (Umlazi)*	162	4	2.5%	132	37	28%
	*Umzinyathi*	226	2	0.9%	126	52	41%
	*Uthukela*	158	0	0.0%	124	22	19%
	*Zululand*	236	14	5.9%	161	43	25%

For preschool-aged children, the age ranged from 1 to 6 years with a mean age of 5 years. At least one class per grade was taken to represent PSAC or SAC. The total number varied from school to school. Eight primary schools and 8 preschools were screened from each district. Due to COVID-19 regulations, the program faced challenges in getting more participants because schools operated on a half class basis. It was also not possible to cover all the schools that had been sampled leading to some districts having less than eight schools being screened. The highest number of participants came from Amajuba (*n* = 261–13%) followed by Zululand (*n* = 236 — 11.6%) and uMzinyathi (*n* = 226 — 11.3%). The lowest was from iLembe district with 124 participants in total.

### Comparing Schistosoma haematobium prevalence between PSAC and SAC

Out of 1176 school-age children screened, 49 (4.2%) were positive for *S. haematobium* with a mean of 52.6 eggs/10 ml. Out of the 824 preschool-aged children screened, only 3 (0.4%) were infected with *S. haematobium* with a mean of 13.2 eggs/10 ml. The intensity range in SAC was 1–250 eggs/10 ml and 8–35 eggs/10 ml in PSAC.

**Fig. 1:**
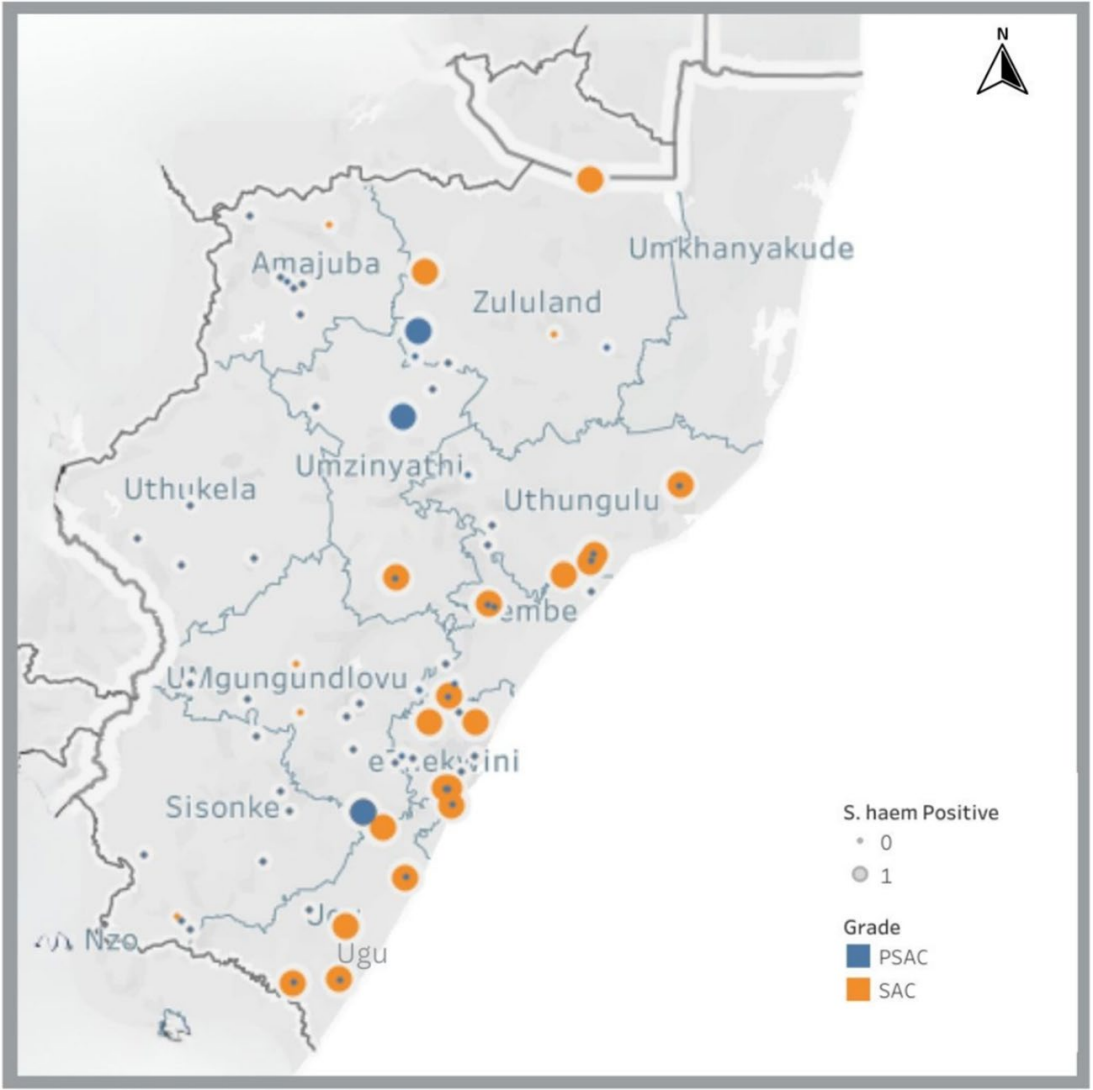
The distribution of infections in the district, *S. haematobium* infections in primary schools are shown in yellow, and preschool infections are shown using the blue dots. The smaller dots show study sites with zero records of infection; some of them appear to be in one colour because two dots are sitting on top of each other; these are schools with both SAC and PSAC

*S. haematobium* infections were more prevalent in coastal districts such as eThekwini, Ugu and iLembe (Fig. 1). For primary school-aged children, *S. haematobium* was more prevalent in rural schools than in urban areas. For example, Zululand, a rural district had a prevalence of 9.3%, almost double that of Umlazi, an urban district with 4.4%. Only three primary school children (all boys) had visible haematuria and none for PSAC. There was no gross haematuria in urban districts for both PSAC and SAC. Table 3 shows details on the prevalence and infection intensity of *S. haematobium* among the two comparison groups.

**Table 3:** Prevalence of *S. haematobium* infections among the two comparison groups (PSAC and SAC)

District	School-age children				Preschool-aged children			
	Total screened (*n*)	*S. haem* + (*n*)	*S. heam* prev (%)	Mean intensisy	Total screened (*n*)	*S. haem P* + (*n*)	*S. heam* prev (%)	Mean intensity
Amajuba	119	0	0.0%	0.00	142	0	0.0%	0.00
iLembe	89	4	4.5%	3.28	35	0	0.0%	0.00
King Cetshwayo	111	5	4.5%	15.92	59	0	0.0%	0.00
Ethekwini — Pinetown	142	3	2.1%	1.48	58	0	0.0%	0.00
Ethekwini — Umlazi	91	4	4.4%	0.55	71	0	0.0%	0.00
Sisonke	85	0	0.0%	0.00	66	0	0.0%	0.00
Ugu	114	15	13.2%	9.59	46	0	0.0%	0.00
Umgungundlovu	75	4	5.3%	2.68	77	1	1.3%	0.08
Umzinyathi	127	1	0.8%	0.32	99	1	1.0%	0.18
Uthukela	90	0	0.0%	0.00	68	0	0.0%	0.00
Zululand	133	13	9.3%	10.33	103	1	1.0%	0.08
Total	1176	49	4.2%		824	3	0.4%	

### Chi-square test of correlation

The results from the chi^2^ test showed a significant difference in the prevalence of *S. haematobium* among PSAC and SAC. The chi-square test shows that there is a statistically significant relationship between grade and *S. haem* positive, *χ*^2^(1) = 27.67, *p* = < 0.001, as seen in Table 4. A Fisher exact test was performed between grade and *S. haem* positive, showing a statistically significant relationship between grade and *S. haem* positive, *p* = < 0.001. The calculated *p*-value of < 0.001 was lower than the defined significance level of 5% (see Table 4). The chi^2^ test was therefore significant, and the null hypothesis was rejected. Simply, there was an increase in infection with every increase in the grade of the children. A child was more likely to get infected with *S. haematobium*, if they were SAC than if they were PSAC.

**Table 4:** Chi^2^ test results determining the correlation between *S. haematobium* infection and grade (PSAC/SAC)

		S. haem positive		
		*0*	*1*	Total
Grade	PSAC	821	3	824
	SAC	1127	49	1176
	Total	1948	52	2000
	Chi^2^	*df*	*p*	
Grade - *S. haem* positive	27.67	1	<.001	
	*p*			
Left-sided	1			
Two-sided	< 0.001			
Right-sided	<.001			
	C			
Grade — *S. haem* positive	0.17			

### Logistic regression test

The study examined the impact of grade, sex and age on *Schistosoma haematobium* infection (Table 5). We found that being SAC increased the odds of *S. haematobium* infection by 1.07, with an odds ratio of 2.92. This means that the odds of SAC being positive were 2.92 times as likely compared to PSAC. This, however, was not statistically significant (*p* = 0.174). Being female decreased the odds of *S. haematobium* infection by − 0.01, with an odds ratio of 0.99. This means that the odds of girls being positive of *S. haematobium* were 0.99 times as likely compared to boys. This analysis was also not statistically significant (*p* = 0.979). Our findings (in Table 5) show that age increased the odds of *S. haematobium* infection by 0.38, with an odds ratio of 1.47, suggesting a 46.75% increase in the odds of infection with each additional unit of age (*p* = 0.005). The *p*-value of 0.005 is below the conventional 0.05 threshold, indicating that impact of age on *S. haematobium* infection was statistically significant at the 5% level.

**Table 5:** The results of a binary logistic regression analysis, which looks at how grade, sex, age influenced the likelihood of a *Schistosoma haematobium* infection

	Coefficient *B*	Standard error	*z*	*p*	Odds ratio	95% conf. interval
Constant	− 7.58	0.94	8.05	< 0.001	0	0–0
Grade SAC	1.07	0.79	1.36	0.174	2.92	0.62–13.68
Sex F	− 0.01	0.28	0.03	0.979	0.99	0.57–1.73
Age	0.38	0.14	2.79	0.005	1.47	1.12–1.92

### Comparing STH prevalence between PSAC and SAC in KwaZulu-Natal Province

A total of 816 SAC and 459 PSAC children provided stool samples for examination. The total number of STH infections among the study participants who provided a stool sample was 281 (22%), out of which 91 were PSACs and 190 SACs. The prevalence of STH in PSAC was 20% and that of SAC was 23%. There was no statistical difference between the two groups; the chi-square test showed that the difference in STH prevalence between PSACs and SACs was not statistically significant (chi-square value of 2.889, *p* = 0.089) implying that, unlike in the case of *S. haematobium*, the prevalence of STHs among SACs and PSACs was similar.

The distribution of STH detected by species was *T. trichiura* 18 (1.4%), *A. lumbricoides* 116 (9%), Taenia 197 (15.3%), Pinworm (*Enterobius vermicularis*) 11 (0.8%), Mansoni 1 (0.07%) and Hookworm species 1 (0.07%). Most of infections appeared together as co-infections. STH infections were not significantly associated with SAC/PSAC for Taenia (*p* > 0.344), 3.136 (*p* > 0.07) for Ascaris, 2.955 (*p* > 0.086) for Trichuria, 0.687 (*p* > 0.407) for Pinworm and 0.562 (*p* > 0.453) for Hookworms.

### Distribution of STH infections in KZN

The highest STH prevalence (52%) is in Pinetown, followed by uMzinyathi (41%), uMlazi (28%) and Zululand (25%), as shown in Table 6. The lowest was uMgungundlovu and iLembe districts at 0.7%, 0.7% and 2.5%, respectively. The highest prevalence of STH for both SAC and PSAC were recorded in Pinetown urban district, with SAC infections being the highest at 44.4% prevalence in the district. There was considerable variation in prevalence between districts ranging from 13 to 44.4%, with very small differences between PSAC and SAC. Refer to Table 6 for more information on STH prevalence per district. The Taenia species had the highest prevalence (20.5% in SAC and 23.4 PSAC) in both groups and all the districts, followed by *Ascaris lumbricoides*. In all the STH infections, Taenia contributed to 72.8% in both groups.

**Table 6:** The prevalence of STH infections among the two comparison groups (PSAC and SAC)

	School-age children											Preschool-aged children						
	STH samples	STH Positive	STH Prevalence	Ascaris	Hookworm	Mansoni	Pinworm	Taenia	Tricuris	STH samples	STH Positive	STH Prevalence	Ascaris	Hookworm	Mansoni	Pinworm	Taenia	Tricuris
Amajuba	42	7	17%	7	0	0	0	0	0	58	7	12%	4	0	1	0	1	0
iLembe	61	5	8%	2	0	0	0	5	0	21	2	10%	1	0	0	0	2	0
King Cetshwayo	88	10	11%	2	0	0	0	8	0	42	8	19%	3	0	0	0	7	0
Thekwini — Pinetown	93	50	54%	29	0	0	3	30	10	29	14	48%	12	0	0	4	6	0
Thekwini — Umlazi	75	26	35%	10	0	0	3	19	2	57	11	19%	3	0	0	0	8	2
Sisonke	63	5	8%	1	0	0	0	4	0	46	5	11%	1	0	0	0	4	0
Ugu	95	7	7%	5	1	0	1	3	0	29	5	17%	2	0	0	0	4	0
Umgungundlovu	41	1	3%	1	0	0	0	1	0	27	1	4%	1	0	0	0	0	0
Umzinyathi	85	35	41%	4	0	0	0	33	3	41	17	41%	3	0	0	0	14	1
Uthukela	74	16	18%	1	0	0	0	13	0	38	8	21%	0	0	0	0	8	0
Zululand	98	28	31%	21	0	0	0	16	0	67	13	19%	3	0	0	0	11	0
**Total**	815	190	23%	83	1	0	7	132	15	455	91	20%	33	0	1	4	65	3

### STH Intensity levels

STH intensity levels for Taenia, Ascaris and Pinworm are shown in Table 7, indicating insignificant differences between PSACs and SACs. The mean intensity for *A. lumbricoides* was 3280 eggs/g and 5810 eggs/g among PSACs and SACs, respectively; mean intensity for Taenia was 380 and 530 eggs/g in PSACs and SACs, respectively. SAC had lower infection intensity of *A. lumbricoides* than PSAC but a higher intensity of Taenia and *Tricuris tricuria* than PSAC. There were no overall differences in infection burden between both groups; they had a high infection burden of *A. lumbricoides* and Taenia and moderate infections of *Trichuris tricuria*. Both urban and rural districts had similar distribution pattern of the STH burden with no differences between boys and girls.

**Table 7:** The STH intensity levels for the 3 species that were frequently recorded; the table also shows the statistical differences between PSACs and SACs

STH species	Geometrical Mean intensity		*t*-Test		Intensity level	
	PSAC	SAC	*F*	*p* value	PSAC	SAC
Taenia	380	530	0.268	0.775	Light	Light
Ascaris	3280	5810	3.481	0.208	Moderate	Heavy
Pinworm	7210	260	16.508	0.231	Heavy	Light

## Discussion

This study examined the prevalence and intensity of schistosomiasis (*S. haematobium*) infection among primary school-aged children (SAC) and preschool-aged children (PSAC) in KwaZulu-Natal Province, South Africa. The study found significant variation in *S. haematobium* infection across different districts, with Zululand reporting the highest prevalence at 9.3%. The study also found that older children were at a greater risk of *S. haematobium* infection, which tends to increase with age due to prolonged exposure to contaminated water sources. The study also found that SAC had a higher prevalence of *S. haematobium*, with 4.2% testing positive for the disease. The study also found a highly localized distribution of the disease, with certain areas being more conducive to schistosomiasis transmission. The study also found a relatively high burden of soil-transmitted helminth (STH) infections among both PSAC and SAC, with Pinetown having the highest prevalence.

### Prevalence of schistosomiasis infection

The findings of this study provide valuable insights into the prevalence and intensity of schistosomiasis (*S. haematobium*) infection among primary school-aged children (SAC) and preschool-aged children (PSAC) in Kwa Zulu Natal Province, South Africa. Our study reveals a considerable variation in the prevalence of S. haematobium infection across different districts, with Ugu district and Zululand reporting the highest prevalence at 9.4% and 5.9%, respectively. Other districts have 0% prevalence such as Uthukela, Sisonke and Amajuba. These districts (Uthukela, Sisonke and Amajuba) are furthest from the coastline and are nestled in mountainous regions, hence the low infection rates due to absence of favourable conditions for the host snails. The districts with high prevalence (Ugu, Zululand and Thekwini) lie in areas where rivers become slow as they approach the ocean; this often provides a good environment for snails to breed with the addition of weeds and marshy waters. These districts are also areas with population settlements. This regional heterogeneity underscores the complex dynamics of schistosomiasis transmission, which are influenced by factors such as local water contact patterns, sanitation and access to healthcare (Mari et al. 2017).

The prevalence of SAC was higher than that of PSAC, suggesting that older children are at a greater risk of *S. haematobium* infection. This is consistent with previous research highlighting that the risk of schistosomiasis tends to increase with age due to prolonged exposure to contaminated water sources (Verjee 2019). It is, however, important to note that contrary to previous thinking that under five prevalence of schistosomiasis was not significant (Stothard and Gabrielli 2007), our study revealed worrying prevalence and intensities.

Among SAC, 4.2% tested positive for *S. haematobium*, with a mean intensity of 51.6 eggs per 10 ml of urine. The presence of visible hematuria, predominantly in primary school-aged boys, serves as a significant clinical indicator of *S. haematobium* infection. Notably, no cases of visible haematuria were observed among PSAC, highlighting a potential difference in disease presentation between the two age groups. This age-related difference in symptomatology may be attributed to variations in the duration and intensity of water contact activities among children of different age groups.

*S. haematobium* infections among PSAC were detected in 2 out of the 10 districts surveyed. Among the SAC, it was detected in 7 out of 10 districts surveyed. This finding suggests a highly localized distribution of the disease, with certain areas being more conducive to schistosomiasis transmission. Our findings on the distribution of *S. haematobium* infection in the province corroborate with findings from the study by Nwoko et al. 2023 on the distribution of human schistosome transmitting snails (*Balinus globosus*) in the province. They found human schistosome transmitting snails in 8 districts out of 11 (Nwoko, Manyangadze et al. 2023).

The statistical analysis revealed significant differences in infection rates between SAC and PSAC in districts where both age groups were affected, particularly in Zululand, which exhibited the highest S. haematobium prevalence. This underscores the importance of localized control strategies, as infection dynamics can vary widely even within the same province. Importantly the low infection intensities highlight the need for more sensitive diagnostic measures, since light infections may not be detected (Utzinger et al. 2015; Rubaba et al. 2018).

### Prevalence of STH infection

The study also investigated the prevalence and intensity of soil-transmitted helminth (STH) infections, revealing a relatively high burden of STH infections among both PSAC and SAC. The overall prevalence rates of 19.8% in PSAC and 23.3% in SAC indicate that STH infections remain a significant public health concern in the study area.

Pinetown (a suburb district of eThekwini) had the highest prevalence of STH infections in both age groups, with SAC exhibiting a slightly higher prevalence at 54%, compared to 48% for PSAC. This was unexpected in an urban district; however, the high prevalence was contributed by two schools which are located close to informal settlements, where there is poor sanitation and poor living conditions. This disparity in infection rates may be linked to differences in hygiene and sanitation practices, highlighting the need for targeted interventions in urban areas.

Among the STH infections, Taenia was the most prevalent, affecting approximately 20.5% of SAC and 23.4% of PSAC. This species accounted for a substantial proportion of the overall STH burden in both age groups. The prevalence of Taenia infections suggests a potential zoonotic transmission, emphasizing the importance of a One Health approach in addressing STH infections in the study area. Wildlife and animals have been shown to be reservoirs of Taenia that has been linked to human infection (Deplazes et al. 2019).

At the provincial level, no significant difference in STH infection burden was observed between SAC and PSAC, indicating that both age groups share a similar risk of STH infections. However, at the district level, variations in infection rates were noted, with Pinetown and uMzinyathi displaying statistically significant distinctions between PSAC and SAC. These district-level variations may be attributed to differences in environmental and socioeconomic factors that influence STH transmission. Pinetown had a high number of informal settlements leading to poor sanitation and hygiene which generally increase STH infections. UMzinyathi also had socio-economic problems which included poor access to water and sanitation facilities.

### Schistosomiasis Intensity and STH Infection Intensity

This study also evaluated the intensity of schistosomiasis and STH infections in both PSAC and SAC. Notably, the mean intensity of *S. haematobium* infection was lower in PSAC (13.2 eggs per 10 ml) compared to SAC (52.6 eggs per 10 ml). The broader range of intensity observed in SAC, ranging from 1 to 250 eggs per 10 ml, highlights the potential for more severe infections among primary school-aged children. Furthermore, the presence of gross haematuria solely in SAC, particularly in the high-prevalence district of Zululand, underscores the clinical severity of schistosomiasis in this age group.

In contrast, STH infections, particularly *A. lumbricoides*, displayed higher mean intensity in PSAC (5810 eggs/g) compared to SAC (3280 eggs/g). However, SAC exhibited higher intensities of Taenia and *Trichuris trichiura* infections. These differences in infection intensity between age groups may be due to factors such as exposure patterns, immunity and host susceptibility.

It is worth noting that the prevalence in this study was lower than those previously found in the same province (Kabuyaya et al. 2017; Manyangadze et al. 2021), and the lower prevalence may be due to preceding droughts. Prevalence of infection generally decreases during prolonged droughts over years due to declining snail populations and rise again after wet spells (Rubaba et al. 2016).

### Study’s contributions and potential implications

The findings of this study have several important implications for public health policy and future research efforts. Firstly, our results underscore the importance of tailored interventions for schistosomiasis and STH control, considering the local epidemiological context. Geographical variations in disease prevalence and intensity within KwaZulu-Natal Province necessitate region-specific strategies to maximize the impact of control programs. As many African counties with low endemicity move towards elimination of schistosomiasis, there is a need for targeted control of infected individuals or regions to avoid development of resistance to the only available drug praziquantel (Tchuenté et al. 2017).

Secondly, the observed high prevalence and intensity of STH infections among both PSAC and SAC emphasize the urgency of integrated STH control measures, including regular deworming, hygiene education and improved sanitation facilities in schools and communities. These measures should be extended to preschools, as our study highlights that PSAC are equally susceptible to STH infections.

Lastly, the absence of *S. haematobium* infections in several provinces among PSAC raises questions about the need for specific intervention strategies targeting this age group. Future research should focus on developing diagnostic tools suitable for PSAC, as well as evaluating the feasibility and effectiveness of treatment programs for this vulnerable population.

This study provides critical insights into the prevalence and intensity of schistosomiasis and STH infections in PSAC and SAC within KwaZulu-Natal Province. These findings contribute to the evidence base for informed public health policies and underscore the need for region-specific control measures and further research to address the neglected tropical diseases affecting young children in South Africa. Our study is opportune in that South Africa still has to roll out its MDA programme; therefore, the results will provide good baseline data for the programme.

## Limitations

Our study was meant to cover the whole of KwaZulu-Natal, but because of COVID-19 restrictions and poor enrolments for PSAC, we were not able to collect data in some districts and in some schools.

One of the limitations of our study was sample representativeness; our sample was calculated at the provincial level, where we chose ten schools per district to represent all the schools in the province. The limitation is that while the sample gives a true prevalence at the provincial level; it does not represent prevalence accurately at the district level since population and number of schools vary per district. As a result, our findings may not paint a true picture of the prevalence at district level, but they show existence of infection in a district. The other implication is that in areas where we found infections, the prevalence may be higher than what we recorded. This necessitates further research in the districts which recorded infections to determine the exact extant of the problem.

In terms of data collection, the most significant limitation was the low stool sample supply. Children aged 5 years and below often struggled to produce stool samples, while the older (school-aged children) did not have much of a problem. Eventually we had a low stool sample count compared to urine. This was, however, rectified using our statistics models that allowed us to adjust the level of statistical confidence based on the samples received.

Another limitation was the difference in sample size between PSAC and SAC groups — the PSAC group had fewer participants. This difference was due to COVID-19 associated constraints encountered in the field such as fewer ECD centres, compared to primary schools, and low enrolment of preschool aged learners in schools offering ECD education. To mitigate this disparity in sample size, specific statistical methods such as chi^2^ test and logistic regression analysis were employed, and the analysis focused on identifying clear effect sizes. The results of the PSAC group were interpreted with caution, acknowledging potential reduced statistical power compared to the SAC group.

## Conclusion

In light of the pressing need to address the burden of schistosomiasis and soil-transmitted helminth (STH) infections in children below the age of 5, our study bridged a critical knowledge gap by comparing the prevalence and intensity of these infections between preschool-aged children (PSAC) and primary school-aged children (SAC) in KwaZulu-Natal Province, South Africa. Our study revealed considerable variation in the prevalence of *S. haematobium* infection in SAC and PSAC across different districts. The overall prevalence of SAC was higher than that of PSAC, suggesting that older children are at a greater risk of *S. haematobium* infection. However, the study proved that a real burden existed among PSACS which needs to be addressed. While no significant difference in STH infection burden was observed between SAC and PSAC at the provincial level, district-level variations were prominent. Notably, the mean intensity of *S. haematobium* infection was lower in PSAC compared to SAC. In contrast, STH infections, particularly *A. lumbricoides*, displayed higher mean intensity in PSAC compared to SAC. However, SAC exhibited higher intensities of Taenia and *Trichuris trichiura* infections. The study also revealed a relatively high burden of STH infections among both PSAC and SAC.

Clinical trial number: Not applicable.

## Data Availability

No datasets were generated or analysed during the current study.
